# Diatom-Bacteria Interactions Modulate the Composition and Productivity of Benthic Diatom Biofilms

**DOI:** 10.3389/fmicb.2019.01255

**Published:** 2019-06-05

**Authors:** Coco Koedooder, Willem Stock, Anne Willems, Sven Mangelinckx, Marleen De Troch, Wim Vyverman, Koen Sabbe

**Affiliations:** ^1^Laboratory of Protistology and Aquatic Ecology, Department of Biology, Ghent University, Ghent, Belgium; ^2^Laboratory of Microbiology, Department of Biochemistry and Microbiology, Ghent University, Ghent, Belgium; ^3^SynBioC Research Group, Department of Green Chemistry and Technology, Faculty of Bioscience Engineering, Ghent University, Ghent, Belgium; ^4^Marine Biology, Department of Biology, Ghent University, Ghent, Belgium

**Keywords:** cross-kingdom interactions, diversity–productivity relationship, microphytobenthos, algae-bacteria relationship, biofilm interactions

## Abstract

Benthic diatoms are dominant primary producers in intertidal mudflats and constitute a major source of organic carbon to consumers and decomposers residing within these ecosystems. They typically form biofilms whose species richness, community composition and productivity can vary in response to environmental drivers and their interactions with other organisms (e.g., grazers). Here, we investigated whether bacteria can affect diatom community composition and *vice versa*, and how this could influence the biodiversity-productivity relation. Using axenic experimental communities with three common benthic diatoms (*Cylindrotheca closterium*, *Navicula phyllepta*, and *Seminavis robusta*), we observed an increase in algal biomass production in diatom co-cultures in comparison to monocultures. The presence of bacteria decreased the productivity of diatom monocultures while bacteria did not seem to affect the overall productivity of diatoms grown in co-cultures. The effect of bacteria on diatom growth, however, appeared to be species-specific, resulting in compositional shifts when different diatom species were grown together. The effect of the diatoms on the bacteria also proved to be species-specific as each diatom species developed a bacterial community that differed in its composition. Together, our results suggest that interactions between bacteria and diatoms residing in mudflats are a key factor in the structuring of the benthic microbial community composition and the overall functioning of that community.

## Introduction

Intertidal mudflats, found predominantly along estuaries and sea inlets, are highly productive ecosystems (Underwood and Kromkamp, 1999). Their productivity is in part due to benthic microalgal biofilms found on the surface sediments (Decho, 2000; Hochard et al., 2010), with benthic diatoms often being one of the dominant primary producers (Admiraal et al., 1984; MacIntyre et al., 1996; Underwood and Kromkamp, 1999; Bolhuis et al., 2013). These biofilms strongly modulate nutrient fluxes (Cook et al., 2009; Hochard et al., 2010) and provide copious amounts of autochthonously fixed carbon to successive trophic levels within the mudflat (Moerdijk-Poortvliet et al., 2018). The productivity of benthic biofilms, dominated by diatoms, depends on the diatom community composition and structure (Brotas and Plante-Cuny, 2003; Underwood et al., 2005) which in turn is dependent on a combination of both abiotic and biotic factors. Although the variable distribution of diatoms along estuarine gradients can partially be explained by the species-specific tolerances of diatom species for environmental conditions such as salinity and nutrient concentrations (Thornton et al., 2002; Forster et al., 2006; Ribeiro et al., 2013; Sawai et al., 2016; Desianti et al., 2017), biotic factors, such as competition and niche differentiation between different diatom species (Vanelslander et al., 2009) as well as the presence of bacteria (D’Costa and Anil, 2011), can also affect the diatom community structure and composition. There is as yet, however, no overall consensus on the exact relation between primary productivity and algal biodiversity of intertidal benthic biofilms. The biodiversity-productivity relation was shown to vary across sites (Forster et al., 2006) and could be either negative or positive (Forster et al., 2006; Vanelslander et al., 2009). Negative biodiversity effects on productivity are often attributed to competition or chemical interference (i.e., allelopathy) while positive biodiversity effects are the result of selection and/or complementarity effects (Loreau and Hector, 2001). A positive selection effect occurs due to the dominance of highly productive species while a complementarity effect increases productivity by enhancing the use of the available resources as a result of positive interactions or niche differentiation. This study investigates the various interactions taking place within a benthic diatom community and whether the presence of bacteria impacts the community regarding species composition and productivity and *vice versa*.

Due to the confined space within the matrix of marine benthic biofilms, interactions between the diatoms, and other organisms become inevitable (Clarke, 2016). The excretion of exopolymeric substances (EPS) by diatoms, for example, serves as an important carbon source that can be utilized by the residing benthic organisms (Middelburg et al., 2000; Bellinger et al., 2009) including heterotrophic bacteria (Middelburg et al., 2000; Taylor et al., 2013; Durham et al., 2015) as well as other diatom species (Vanelslander et al., 2009). Since the composition of diatom exudates is both species-specific and dependent on environmental conditions (Durham et al., 2015; Bohórquez et al., 2017), EPS has been suggested to play a major role in determining bacterial community composition and diversity (Haynes et al., 2007; Agogue et al., 2014; Wear et al., 2015; Mühlenbruch et al., 2018). Indeed, studies have shown that diatom species can harbor different associated bacteria (Schafer et al., 2002; Grossart et al., 2005; Doiron et al., 2012; Behringer et al., 2018) and that bacterial community composition in intertidal mudflats strongly co-varies with the composition of the microphytobenthos (Bolhuis et al., 2013; Decleyre et al., 2015; Lavergne et al., 2017).

In turn, the associated bacteria have also been shown to influence diatom growth rates as well as other life cycle features such as sexual reproduction (Grossart, 1999; Amin et al., 2012; Cirri et al., 2018). Despite the general view of heterotrophic bacteria as the primary remineralisers of (e.g., diatom-derived) organic matter (Azam, 1998; Taylor et al., 2013), releasing nutrients back into the environment, the bacterial community can also compete with diatoms for limited nutrients and resources (Thingstad et al., 1993; Grossart, 1999; Havskum et al., 2003; Amin et al., 2012). Other, more specific, interactions have also been observed, such as the ability of strains to produce vitamins (i.e., cobalamin) for auxotrophic diatoms (Haines and Guillard, 1974; Croft et al., 2005), as well as negative interactions such as the production of algicidal metabolites and the induction of diatom cell lysis (Furusawa et al., 2003; Paul and Pohnert, 2011). These effects can be highly specific, with some bacteria having the ability to stimulate the growth of one diatom whilst inhibiting the growth of another (Grossart, 1999; Jung et al., 2008; Paul and Pohnert, 2011; Sison-Mangus et al., 2014).

As many of the above-mentioned studies focused on one-to-one interactions between diatoms and bacteria, they do not reflect the ecological complexity found in nature. Bigalke and Pohnert (2019), for example, recently illustrated that a more complex diatom community reacts differently to bacteria than the individual species did. While biotic and abiotic factors could promote the presence of a potentially harmful bacterial community (Marzinelli et al., 2018) the host-associated bacterial community can also play a role in its resistance and tolerance toward a new or stressful environment (Dittami et al., 2016). Studies like these, stress that the oversimplification of experiments can prevent further insight into relevant ecological interactions. Whether a bacterial community can influence community functioning in terms of productivity and the outcome of interspecific interactions amongst co-occurring biofilm diatom species has to date not been tested. Although D’Costa and Anil (2011) have shown that diatom community structure is related to bacterial community composition, it was not determined whether and how diatom-bacteria associations could affect changes in diatom productivity and interactions. Recent work on plants and macroalgae, suggests that feedback mechanisms between hosts and their specific bacterial community can ultimately determine the outcome of competition amongst host species (Dittami et al., 2016; Gribben et al., 2017; Hortal et al., 2017; Lekberg et al., 2018; Marzinelli et al., 2018).

Using different benthic diatom species grown at three diversity levels (monoculture vs. 2- and 3-species co-cultures) in the presence or absence of a natural bacterial inoculum, we investigated whether bacteria can change the structure and productivity of simple diatom communities. We hypothesize these interkingdom interactions are species-specific and that certain bacteria promote the growth of selected diatoms and *vice versa*. We therefore predict that the interactions between diatoms and bacteria will change the outcome of competition amongst the different diatom species, favoring the diatom species that benefit the most from the presence of bacteria. This, in turn, could further influence diatom productivity. Finally, we tested whether diatoms can also affect bacterial diversity, predicting an increase in bacterial diversity with increasing diatom diversity and productivity.

## Materials and Methods

### Experimental Setup

The marine benthic diatoms *Cylindrotheca closterium*, *Navicula phyllepta*, and *Seminavis robusta* were obtained from the BCCM-DCG culture collection (Supplementary Table S1). The three strains were originally not isolated from the same location, but these three diatom species have been observed to co-occur, making them ecologically relevant for this study. Diatoms were cultivated in artificial sea water (ASW; Tropic Marin Bio-Actif Salt) that was enriched with 0.08 g/L NaHCO_3_ and Guillard’s Marine Water Enrichment Solution (F/2; Sigma) according to the manufacturer’s instructions. Throughout the experiment, cultures were grown in 12:12 h light-dark cycles using cool fluorescent white light (20–25 μmol photons/s/m^2^) and maintained at 18°C.

Diatoms were made axenic (free of bacteria) by subjecting them to repeated antibiotic treatments. Every 3 days, the medium of the diatoms was refreshed and the next antibiotic treatment was given to the cultures. This process was repeated at least three times. Antibiotic mixes consisted of final concentrations of 100 μg/mL gentamicin, 500 μg/mL streptomycin, and 100 μg/mL neomycin for *C. closterium*; and 500 μg/mL penicillin, 500 μg/mL ampicillin, 100 μg/mL streptomycin, and 50 μg/mL gentamicin (Sigma-Aldrich) for *N. phyllepta* and *S. robusta*. DAPI staining (Shishlyannikov et al., 2011) and plating on Difco Marine agar (BD) was used to confirm axenicity of the diatom cultures after the treatments. Although this study did not specifically test whether the antibiotic treatments resulted in a decline in health or a decreased growth rate for the individual diatom species used within this experiment it was done in the framework of another study (Stock et al., unpublished).

Prior to the start of the experiment, antibiotics in the diatom cultures were washed away by repeated (three times) refreshing of medium with new ASW 3 days after the last antibiotic treatment. The refreshed cultures were diluted to approximately 300, 100, and 85 cells/mL for *S. robusta*, *C. closterium*, and *N. phyllepta*, respectively. Dilutions were based on prior experiments (data not shown), which showed that the coexistence between the diatom species required higher starting densities of *S. robusta*. At equal starting densities, *S. robusta* was rapidly outcompeted due to its lower growth rate.

The bacterial inoculum was obtained from intertidal surface mud collected at the Paulina polder, Westerschelde, NL (2 March, 2016 at 51°21′032′′N, 3°43′574′′ E). This sample was taken 1 day before setting up the experiment. During this time, it was stored at 4°C. On the day of the experiment, 20 mL of ASW was added to ± 10 mL of sediment, vortexed and then filtered (3 μm filter size) to separate the bacteria from larger eukaryotic organisms and particles. 1 mL of the bacterial suspension was frozen (-20°C) for DNA extraction (see below).

The experiments were run in 24-well plates (Greiner Bio-one) with the addition of either 1 mL of bacterial inoculum (non-axenic) or 1 mL of ASW (axenic). 1 mL of diatom suspension was then added to obtain a total volume of 2 mL per well. Specifically, 1 mL of a single diatom, 2 × 0.5 mL of two diatoms or 3 × 0.333 mL of all three diatoms were added to obtain monocultures and 2- and 3-species co-cultures, respectively. F/2 stock solution was supplemented to all wells so that a final nutrient concentration of F/20 (one tenth of F/2) was reached. All different combinations were run in triplicate (*n* = 3), randomized across each plate and grown in the conditions stated above.

Daily biomass estimates were obtained using both pulse amplitude modulation (PAM) fluorometry (IMAGING-PAM M-Series Maxi version, Walz) and cell counts (Stock et al., 2019). Minimal fluorescence (F_0_) was measured (intensity: 6, gain: 3, and frequency: 1) daily, for a period of 7 days. Photographs were taken of each well in triplicate (Nikon Elemental Camera DS-Fi2 with 10x magnification) using an inverted Axiovert 135 Zeiss microscope. Diatoms were manually counted from the pictures (Supplementary Figure S1) and averaged to obtain daily diatom cell counts.

After 1 week, 1 mL of medium was extracted from every well and stored at -20°C for DNA analysis. DNA extractions were conducted according to Muyzer et al. (1993). The library prep and amplification of the 16S rRNA gene, using primers PA_ill and BKL1_III, were done according to D’Hondt et al. (2018). An artificially created mock community was included to benchmark processing variables (Tytgat et al., 2016; Supplementary Table S2).

### Data Analysis and Statistics

#### Diatoms

Growth curves were constructed from diatom cell counts and PAM fluorometry measurements (F_0_) using Excel (Version 15.32). The maximal growth rate (μ_max_) was defined as the highest observed growth rate during the exponential growth phase (the first 4 days as determined after inspection of each individual growth curve) of the experiment and calculated from the slope (LINEST function) after a log2 transformation for every three consecutive days using a moving window. This strategy was applied to both F_0_ and diatom cell counts. A two-way ANOVA (R-version 3.4.1) was used to test for differences in μ_max_ between diatom combinations and in the presence or absence of bacteria. The assumptions of ANOVA regarding homogeneity of variance and normality were, respectively verified through a Levene and Shapiro–Wilk Test. Where appropriate, a Tukey HSD test was applied as *post hoc* test.

Diatom productivity was defined as the increase in the diatom biovolume and calculated by multiplying the μ_max_ obtained from the cell counts with the cell biovolume calculated according to Hillebrand et al. (1999). Productivity was expressed in function of diatom biodiversity. Biodiversity effects were further partitioned into selection and complementarity effects according to Loreau and Hector (2001) in R (version 3.4.1). The partitioning of biodiversity effects was done separately for both axenic and non-axenic cultures.

#### Bacteria

The 300 bp pair-end MiSeq reads were joined and quality-filtered using PEAR (Version 0.9.5). After primer removal, an operational taxonomic unit (OTU) table was constructed by clustering at a 3% divergence level (USearch8). Taxonomic assignment of OTUs was done using MOTHER (Version 1.35.1). Chimeras were removed using the internal check in Usearch whilst mitochondrial and chloroplast reads were removed based on taxonomic assignment. The OTU table was further processed in R (version 3.4.1). To remove potential cross-contamination and sequencing errors, read counts <5 and OTUs that were present in less than three samples were set to 0 (based on 16S mock community data). Relative abundances were derived from the constructed OTU table. Rare OTUs (<1% of number of reads/sample) were removed. A PERMANOVA was run using the Adonis function (Vegan Package 2.4-3) between all diatom combinations and treatments. Significance values were based on 1000 permutations. A constrained correspondence analysis (ANOVA-CCA, Vegan Package 2.4-3) was performed with different diatom combinations set as Boolean variables to identify the influence of the different diatom species on the bacterial community composition. Finally, a Simper Analysis (10000 permutations, Vegan Package 2.4-3) was run to identify the discriminating bacterial OTUs between the diatom monocultures. Bacterial diversity, expressed as both the predicted number of OTUs when rarefying all samples to the same depth and the Simpson’s index, was also linearly expressed in function of both diatom productivity and diatom-diversity. This procedure was separately repeated for the most diverse bacterial group, namely the Alphaproteobacteria.

## Results

### Diatom Growth

**FIGURE 1:**
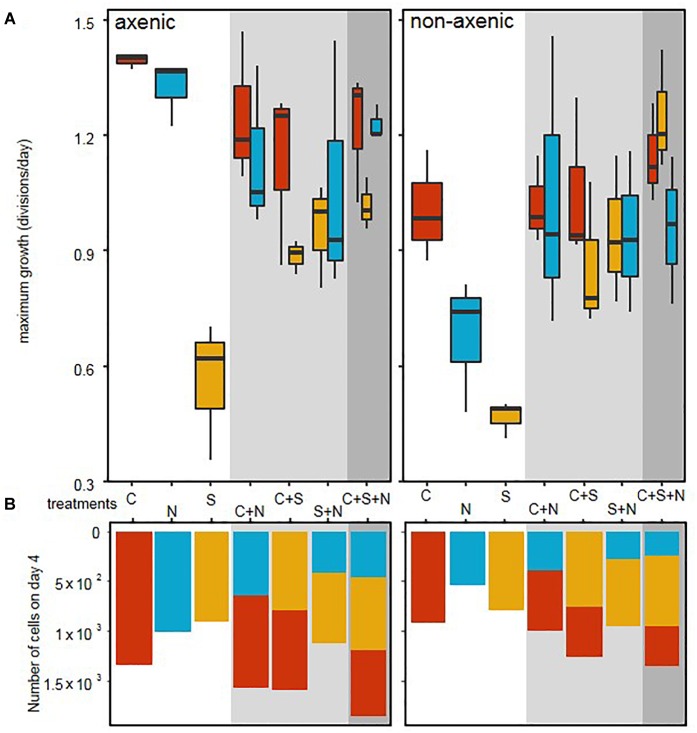
Bacteria cause a shift in diatom growth rates. **(A)** Maximum growth rates, derived from daily cell counts for three different diatom species: *C. closterium* ([C], red), *N. phyllepta* ([N], blue), and *S. robusta* ([S], yellow) in the presence (non-axenic) or absence (axenic) of bacteria. The white, gray and dark gray background, respectively show diatoms grown either in monocultures, diatom pairs or the three diatom-species combined. **(B)** The number of diatom cells per well on the fourth and last day are shown using the same color code as above.

In axenic conditions, *C. closterium* and *N. phyllepta* displayed comparably high μ_max_ in monocultures while *S. robusta* grew considerably slower (Figure 1 and Table 1). The presence of other diatoms did not influence the μ_max_ of *C. closterium* (*p* > 0.05) and *N. phyllepta* (*p* > 0.05) but significantly increased (*p* < 0.001) the μ_max_ of *S. robusta* (Figure 1A). The addition of a mixed natural bacterial inoculum to the monocultures significantly lowered the μ_max_ of *C. closterium* (*p* = 0.011) and *N. phyllepta* (*p* = 0.007), but not of *S. robusta* (*p* > 0.05) (Figure 1A). In the non-axenic co-cultures, the negative effect of bacteria on the growth of *N. phyllepta* and *C. closterium* seemed slightly alleviated by the presence of other diatoms (albeit not significantly), although the total cell numbers never exceeded those of their axenic counterparts (Figure 1B). The growth rate of *S. robusta* was again significantly higher (*p* ≤ 0.003) in mixed diatom cultures and in the presence of the two other diatom species, *S. robusta* was the fastest grower (Figure 1A). These species-specific changes in growth rates due to the presence of bacteria resulted in compositional shifts in the diatom co-cultures: the relative abundance of *S. robusta* was higher when bacteria were present, mainly at the expense of *C. closterium* (Supplementary Figures S3, S4). Similar trends were observed for the μ_max_ derived from daily fluorescence measurements (Supplementary Figure S5 and Supplementary Table S3) although PAM measurements did not allow separation of biomass increase between diatom species grown in co-cultures. Altogether, changes in μ_max_, from both cell counts (Figure 1A) or PAM measurements (Supplementary Figure S5) as well as the total biovolume (Supplementary Figure S4) indicate that, while the growth rates of *C. closterium* and *N. phyllepta* were significantly repressed in the presence of bacteria, *S. robusta* was left unaffected while its μ_max_ significantly improved in the presence of other diatom species.

**Table 1:** Statistical analysis of the maximum growth rates derived from individual cell counts for *S. robusta* [S], *C. closterium* [C], and *N. phyllepta* [N].

*Cell Counts*					
***Two-way ANOVA***	**Factor**	**DF**	***F* value**	***p*-value**	**significance**
***C. closterium***	diatom combination	3	0.527	0.670	
	bacteria – axenic	1	8.250	0.011	^∗^
	interaction	3	1.179	0.349	
		16			

***Post hoc***	**Conditions**	**Difference**		***p*-value**	**significance**

	bacteria – axenic	-0.194		0.011	^∗^

***Two-way ANOVA***	**Factor**	**DF**	***F* value**	***p*-value**	**significance**

***S. robusta***	diatom combination	3	20.784	<0.001	^∗∗∗^
	bacteria – axenic	1	0.210	0.653	
	Interaction	3	1.554	0.239	
		16			

***Post hoc***	**Conditions**	**Difference**		***p*-value**	**significance**

	[s] – [s+c]	-0.359		0.002	^∗∗^
	[s] – [s+n]	-0.438		0.003	^∗∗^
	[s] – [s+c+n]	-0.621		<0.001	^∗∗∗^
	[s+n] – [s+c]	-0.078		0.770	
	[s+c] – [s+c+n]	-0.261		0.024	^∗^
	[s+n] – [s+c+n]	-0.183		0.148	

***Two-way ANOVA***	**Factor**	**DF**	***F* value**	***p*-value**	**significance**

***N. phyllepta***	diatom combination	3	0.302	0.824	
	bacteria – axenic	1	9.257	0.008	^∗∗^
	interaction	3	1.807	0.186	
		16			

***Post hoc***	**Conditions**	**Difference**		***p*-value**	**significance**

	bacteria – axenic	-0.285		0.008	^∗∗^

*A two-way ANOVA was conducted testing the differences between the diatom combinations and the presence or absence of bacteria per diatom species. A post hoc test (Tukey Test) was conducted when significant differences were observed from the ANOVA. An asterisk marks results of significant value where ^∗^<0.05, ^∗∗^<0.01, and ^∗∗∗^<0.001.*

### Additive Partitioning of Biodiversity Effects

**FIGURE 2:**
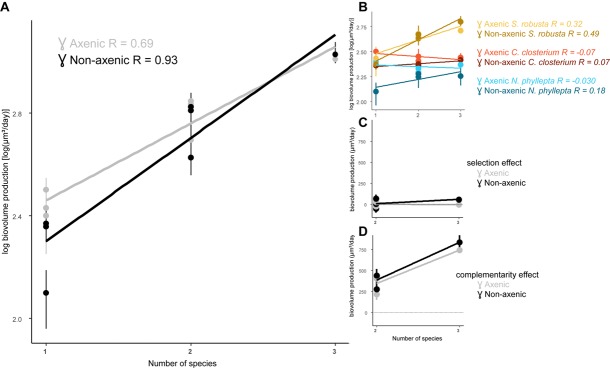
The presence of bacteria steepens the algal diversity-productivity relation. **(A)** Diatom biovolume production in function of species richness and its slope (m) in the presence (non-axenic, black line) or absence of bacteria (axenic, gray line). **(B)** Algal biovolume production per diatom species and its slope for each diatom: *S. robusta* (yellow), *C. closterium* (red), and *N. phyllepta* (blue). The presence or absence of bacteria are, respectively depicted as dark- and light-colored lines. The contribution of the selection and complementarity effects to diversity-productivity relation are, respectively shown in **(C,D)** in the presence (black) or absence (gray) of bacteria.

We determined whether increasing diatom species richness had an impact on productivity, i.e., the algal biomass production, and whether this would change in the presence of bacteria (Figure 2). In general, productivity increased with increasing diatom diversity (*p* < 0.0001) and the presence of bacteria steepened this relationship (*p* = 0.007; Figure 2A). This effect of bacteria can largely be attributed to a strong decrease in productivity in the diatom monocultures when exposed to the bacterial inoculum (*p* = 0.045). In contrast, the bacteria have a reduced negative effect on the diatom species grown in the presence of other diatoms.

Analogous to the results obtained from the diatom growth rates, the productivity of *S. robusta* strongly increased in the presence of other diatoms under axenic conditions, while both *C. closterium* and *N. phyllepta* had lower productivity in the co-cultures (Figure 2B). Adding bacteria switched the observed negative diversity effect in *C. closterium* and *N. phyllepta* to a positive diversity effect, with both species now showing higher productivity in the co-cultures. This effect, however, was not very strong, and was always less pronounced in comparison to *S. robusta* (Figure 2B).

The effect of diatom diversity on the productivity was further partitioned into selection and complementarity effects (Loreau and Hector, 2001; Figure 2C,D). The observed increase in production (*p* < 0.0001) can largely be attributed to positive complementarity effects, which further increased in the presence of bacteria (*p* > 0.05). A positive but very minor selection effect on productivity was also observed in the presence of bacteria (*p* > 0.05).

### Bacteria Community Composition

**FIGURE 3:**
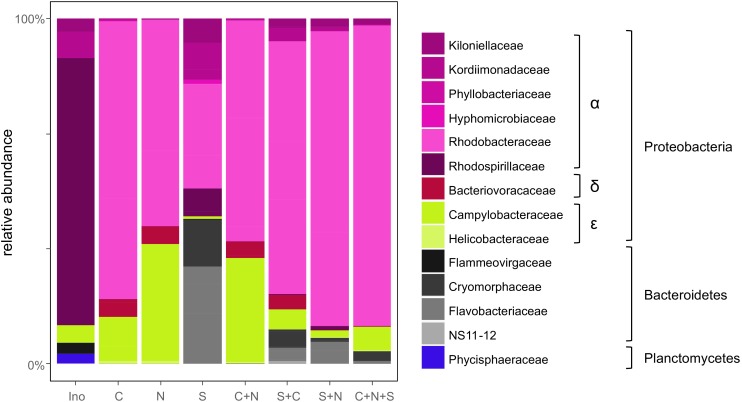
Bacterial community composition follows the diatom community. Relative bacterial abundances present in the original inoculum (Ino) or when grown with either *C. closterium* [C], *N. phyllepta* [N], S. robusta [S], or a combination of these diatoms. The different bacterial orders are depicted using different colors.

After removing non-bacterial and potentially chimeric reads from the 16S rDNA high-throughput sequencing, 8724 ± 5505 (mean ± SD) reads per sample remained. These were assigned to 123 OTUs. Based on the rarefaction curves (Supplementary Figure S2) we determined that the sequencing depth was sufficient to detect the most abundant bacteria present in the algal cultures. The composition of the initial bacterial inoculum was dominated by *Rhodospirillaceae* (41.5% of the inoculum reads) (Figure 3). Following co-cultivation with diatoms over a week, the bacterial communities became dominated by *Rhodobacteraceae*, *Flavobacteriaceae*, and *Campylobacteraceae* both in terms of diversity (32, 18, and 8%, respectively) and relative abundance (68, 6, and 14%, respectively). Multivariate analyses of the data (Supplementary Figure S6) showed a strong differentiation (PERMANOVA: *p* = 0.006) between the bacterial communities associated with the different diatom combinations. The strongest difference was noticeable between the *S. robusta* monocultures and the cultures containing *C. closterium* and *N. phyllepta* (ANOVA-CCA: *p* = 0.01), although there were some minor differences between the latter bacterial communities as well (ANOVA-CCA: *p* > 0.05). The bacterial communities associated with *S. robusta* monocultures had a much higher diversity (H’ = 2.01 ± 0.15 based on the rarefied data) in comparison with the bacterial communities associated with the other diatoms (H’ = 1.11 ± 0.21 and H’ = 0.87 ± 0.09 for *C. closterium* and *N. phyllepta* monocultures, respectively).

Bacterial communities formed in the presence of *S. robusta* typically contained (amongst others) *Alphaproteobacteria* (*Thalassospira* sp., *Roseobacter* sp., and a *Kordiimonadaceae* sp.) and *Bacteroidetes* (*Mangrovimonas* sp. and *Owenweeksia* sp.). Especially the *Mangrovimonas* sp. tended to be highly abundant in *S. robusta* cultures (up to 42%) whilst being almost absent in *C. closterium* and *N. phyllepta* monocultures. In contrast, the bacterial communities of the latter monocultures were dominated (up to 80% of relative abundances) by an *Octadecabacter* species (*Alphaproteobacteria, Rhodobacteraceae*) and to a lesser degree also by an unidentified *Arcobacter* sp. (*Epsilonproteobacteria*). Bacterial diversity in the diatom co-cultures was not higher than in the monocultures (Supplementary Figure S7). Instead, an intermediate community structure was observed which was more similar to bacterial community present in the *N. phyllepta* and/or *C. closterium* monocultures. Finally, no linear relation (*p* > 0.05) was found between bacterial diversity, as a whole or for the *Alphaproteobacteria* separately, and diatom productivity.

## Discussion

Our results show a positive relationship between benthic diatom diversity and productivity in simple experimental diatom communities. Although the biodiversity-productivity relation has been shown to vary for diatoms at varying spatial scales and conditions (Gessner et al., 2004; Giller et al., 2004; Soininen, 2009; Blanco et al., 2012; Baert et al., 2016), our results are in accordance with what was observed previously for marine benthic diatoms (Forster et al., 2006; Vanelslander et al., 2009). According to the biodiversity-productivity relation, the positive effect of diversity on productivity could largely be attributed to complementarity effects (Loreau and Hector, 2001). This indicated that the competition between diatom species was generally smaller than competition within diatom species and was likely the result of the improved usage of the available resources between the diatom species through cross-feeding and facilitation events (Fridley, 2001; Bruno et al., 2003). Although our experimental setup did not allow further analysis of EPS production and utilization, the presence of chemical cross-talk between diatoms, which include changes in the production, release and utilization of organic compounds, seems to be relatively common (Tuchman et al., 2006) and has been shown to stimulate the growth of co-cultured diatoms (Paul et al., 2009; Vanelslander et al., 2009). For example, the spent medium derived from *N. phyllepta*, resulted in a mixotrophic switch that improved the growth rate of a specific *C. closterium* strain, by benefitting from the carbon released by *N. phyllepta* (Vanelslander et al., 2009). The capability of such a mixotrophic lifestyle, however, appears to be strain dependent (Mensens et al., 2015) and was not observed for the *C. closterium* strain used in this study (Audoor *unpublished data*). Regarding our results, a similar process, however, may be occurring in *S. robusta*, whose growth was positively affected by the presence of other diatom species and would require further testing.

Our experimental setup revealed the recruitment of distinct bacterial assemblages from a common bacterial inoculum by individual diatom species, which is in accordance with previous studies (Schafer et al., 2002; Grossart et al., 2005; Jasti et al., 2005; Eigemann et al., 2013; Bagatini et al., 2014; Sison-Mangus et al., 2014). Interestingly, despite an expected increase in substrate and habitat heterogeneity (Kassen et al., 2000; Giller et al., 2004), the combination of different diatom species did not result in a higher bacterial diversity. Neither could a relation be found between primary production and bacterial diversity, either as a whole or within certain taxonomic groups (Horner-Devine et al., 2003; Stein et al., 2014; Raes et al., 2018). Instead, the bacterial community composition of a mixture of diatom species reflected an intermediate combination of the bacteria that were present in the three separate diatom monocultures.

Although *S. robusta* and *N. phyllepta* are more closely related to one another, the bacterial community composition of *N. phyllepta* was more similar to that of *C. closterium*. *S. robusta* harbored a diverse bacterial community in comparison to the bacterial communities of *N. phyllepta* and *C. closterium* which were both dominated by *Octadecabacter* sp., a member of the *Roseobacter* clade, and *Arcobacter* sp. The dominance of both bacteria over the other bacteria, coinciding with a negative impact on the growth rate of two out of the three diatom species, is a possible indicator of a negative and potentially allelopathic effect from these bacteria on other microorganisms (Mayali et al., 2008; Slightom and Buchan, 2009). A literature search revealed that not much is known about marine *Arcobacter* species (Collado and Figueras, 2011), but the genus does include several important mammalian pathogens (Ferreira et al., 2015). Several *Roseobacter* representatives, on the other hand, are known to reduce both algal and bacterial growth (Mayali et al., 2008; Seyedsayamdost et al., 2011; Sharifah and Eguchi, 2011), e.g., through the production of antibiotics (Ruiz-Ponte et al., 1999; Brinkhoff et al., 2004; Wagner-Dobler et al., 2004; Bruhn et al., 2007). Interestingly, the reduced manifestation of *Octadecabacter* and *Arcobacter* in the presence of *S. robusta*, whose growth rate was left unaffected in the presence of bacteria, could further suggest the ability of either *S. robusta* or its associated microbial community to suppress the growth of these bacteria or their potential antibiotic activity.

Although several studies have highlighted the species-specific effects of bacteria on diatom growth (e.g., Grossart, 1999; Amin et al., 2012; Doiron et al., 2012) and diatom community composition (D’Costa and Anil, 2011), our results indicate how these diatom-bacteria interactions may further impact the outcome of diatom-diatom competition and the subsequent effect on productivity. Indeed, few studies have investigated the impact of bacteria-host interactions on community functioning and results appear to differ (Hubbard et al., 1986; Grime et al., 1987; Horner-Devine et al., 2003; Pugnaire et al., 2004; Kardol et al., 2007; Gribben et al., 2017; Hortal et al., 2017; Laforest-Lapointe et al., 2017) and are dependent on the environmental conditions (Callaway et al., 2004; Dittami et al., 2016; Marzinelli et al., 2018). In the absence of bacteria, experimental diatom communities were dominated by the species that showed the highest growth rate in monoculture, i.e., *C. closterium*. In mixed diatom cultures, the addition of bacteria negatively impacted the growth of both *C. closterium* and *N. phyllepta*, leading to the dominance of *S. robusta*. The presence of a bacterial community further altered the algal diversity functioning relationship, steepening the positive relationship found between diatom diversity and productivity as a result of an enhanced complementarity effect. Similar to what has been observed in plants (Lekberg et al., 2018), the most competitive diatoms in our study experienced the most negative bacterial effects which in turn resulted in the bacteria indirectly promoting co-existence amongst the diatom species. Even if our simple experimental setup does not permit further extrapolation to field situations, our findings, using three naturally co-occurring diatom species, call for a broader consideration of the role of benthic microbiota in shaping diatom community structure and function. Our findings fit well within the holobiont concept, which considers both hosts and their microbes as a single integrated unit (Zilber-Rosenberg and Rosenberg, 2008). Although it remains to be shown that co-evolution between diatoms and bacteria is commonplace, as is often stated in this theory (Skillings, 2016), the strong interactions between host-diatoms and their associated bacteria have important implications for the overall diatom fitness and thus their established niche (Vandenkoornhuyse et al., 2015; Kopac and Klassen, 2016) in species-rich natural communities.

## Data Availability

The 16S rRNA raw sequence data were deposited in the NCBI Sequence Read Archive under the accession number PRJNA521472. The count data, growth rates and OTU table can be found on Github (https://github.com/willem-stock/Koedooder-et-al.-2019).

## Author Contributions

CK, KS, and WS designed the study. CK and WS performed the experimental work and analyzed the data. CK and WS wrote the manuscript, which was revised by all authors. KS and WV provided funding for the research. All authors contributed to the discussion of the results.

## Conflict of Interest Statement

The authors declare that the research was conducted in the absence of any commercial or financial relationships that could be construed as a potential conflict of interest.

## Supplementary Material

FIGURE S1 Example of picture-based diatom cell counting. The image shows a mixed diatom culture inoculated with bacteria at day 3. The culture contains *S. robusta* (pink, nr. 1), *C. closterium* (cyan, nr. 2), and *N. phyllepta* (yellow, nr 3). The image size is a fourth of the pictures taken for the cell counts.

FIGURE S2 Rarefaction curve for every sample (before removal of rare OTUs) is shown. Colors indicate the different treatments as displayed.

FIGURE S3 The presence of bacteria causes a shift in the relative proportions of diatoms. The relative proportion of *C. closterium (red)*, *S. robusta (yellow)*, and *N. phyllepta (blue)* grown in pairs or altogether expressed as the number of cells per species to the total number of cells in the well (Left = T_1_, Center = T_4_ in axenic conditions, and Right = T_4_ in non-axenic conditions). Briefly, in axenic conditions an increase in the proportion of *C. closterium* is observed when grown in the presence of *S. robusta*, while this observation was not observed in the presence of bacteria. Instead, *S. robusta* had an increased overall proportion in comparison to *C. closterium*.

FIGURE S4 The change in the total biovolume (μmł) for each of the three diatom species [*C. closterium* (red), *N. phyllepta* (blue), and *S. robusta* (yellow)] 1 day after inoculation (green area) and 4 days after inoculation (purple area). The gray line indicates the biovolumes under non-axenic conditions while the black line specifies those under axenic conditions.

FIGURE S5 PAM measurements depicting the total fluorescence as a proxy for the total maximum growth rates for different combinations of *C. closterium* ([C], red), *N. phyllepta* ([N], blue), and *S. robusta* ([S], yellow) in the presence (non-axenic) or absence of bacteria (axenic). Combinations of multiple diatoms species together have the appropriate colors combined. The white, gray and dark gray background, respectively show diatoms grown in monocultures, diatom pairs or the three diatom-species combined.

FIGURE S6 PCA on the relative abundance of the bacterial community from the original inoculum (black circle) and when grown in the presence of different combinations of *C. closterium* (red), *N. phyllepta* (blue), and *S. robusta* (yellow). Samples with more diatoms present have circles with the appropriate colors combined. Gray arrows indicate the vectors of the bacterial OTUs with a cumulative loading larger than 30% on the first two axes. The bacterial genus or family is indicated depending on the closest hit. The proportion of the variance explained by each axis is indicated next to that axis.

FIGURE S7 Diversity levels of bacteria in the presence of different diatom combinations of *C. closterium* (C, red), *N. phyllepta* (N, blue), and *S. robusta* (S, yellow). Bacterial community diversity was calculated as the average number of predicted OTUs after rarefication of the samples to 781 reads.

TABLE S1 Information on the three different diatom strains used within our experimental setup. More information can be found at the Belgian Co-ordinated Collections of Micro-organisms website using the diatom accession number (http://bccm.belspo.be/catalogues).

TABLE S2 Composition of the mock community. The raw fastq data of the mock is available at NCBI Sequence Read Archive under the accession number SUB5539877. Briefly, the DNA was extracted and amplified for each strain individually and equimolar pooled afterward. The collection number refers to the identifier of the strains in the BCCM/LMG collection (http://bccm.belspo.be/about-us/bccm-lmg; LMG) or the research collection of the Laboratory of Microbiology Ugent (http://lmg.ugent.be; R).

TABLE S3 Statistical analysis of the total maximum growth rates derived from F_0_ PAM values of 24-plate wells harboring different combinations of *S. robusta* [S], *C. closterium* [C], and *N. phyllepta* [N]. A two-way ANOVA was conducted testing the differences between the total growth rate of the diatom combinations (single, paired, or all together) and the presence or absence of bacteria. A *post hoc* (Tukey Test) was further conducted on each of the different diatom combinations. An asterisk marks results of significant value where ^∗^<0.05, ^∗∗^<0.01, and ^∗∗∗^<0.001.
