# Supplementary figures and images for: Diatom-Bacteria Interactions Modulate the Composition and Productivity of Benthic Diatom Biofilms
Source: Front Microbiol. 2019 Jun 5;10:1255. doi: 10.3389/fmicb.2019.01255 (PMC6561236; doi:10.3389/fmicb.2019.01255)

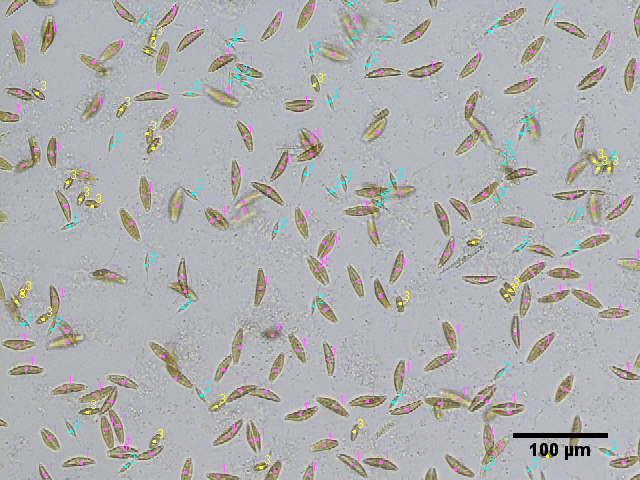

Supplement: FIGURE S1 — Example of picture-based diatom cell counting. The image shows a mixed diatom culture inoculated with bacteria at day 3. The culture contains S. robusta (pink, nr. 1), C. closterium (cyan, nr. 2), and N. phyllepta (yellow, nr 3). The image size is a fourth of the pictures taken for the cell counts. [file Image_1.TIF]

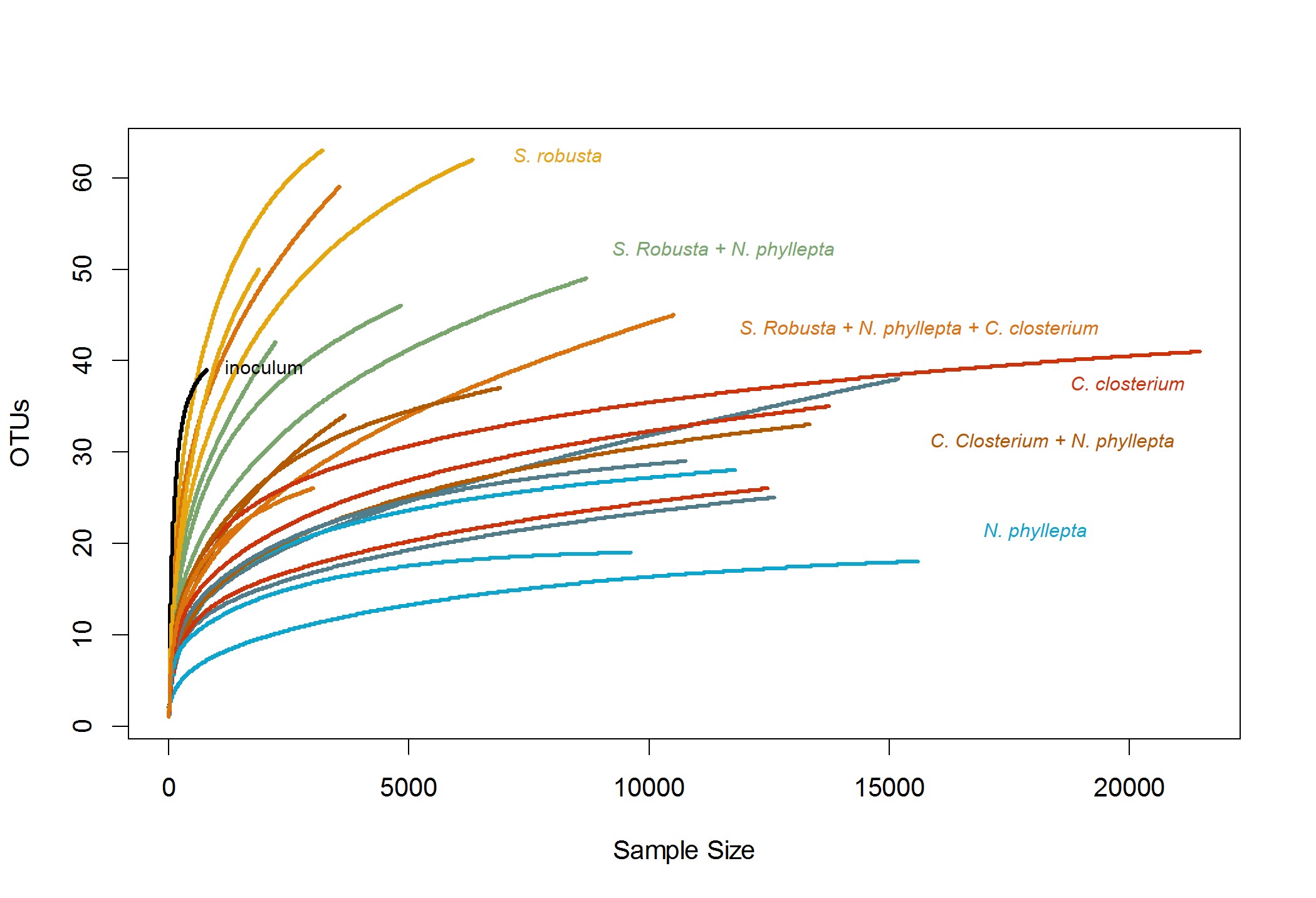

Supplement: FIGURE S2 — Rarefaction curve for every sample (before removal of rare OTUs) is shown. Colors indicate the different treatments as displayed. [file Image_2.JPEG]

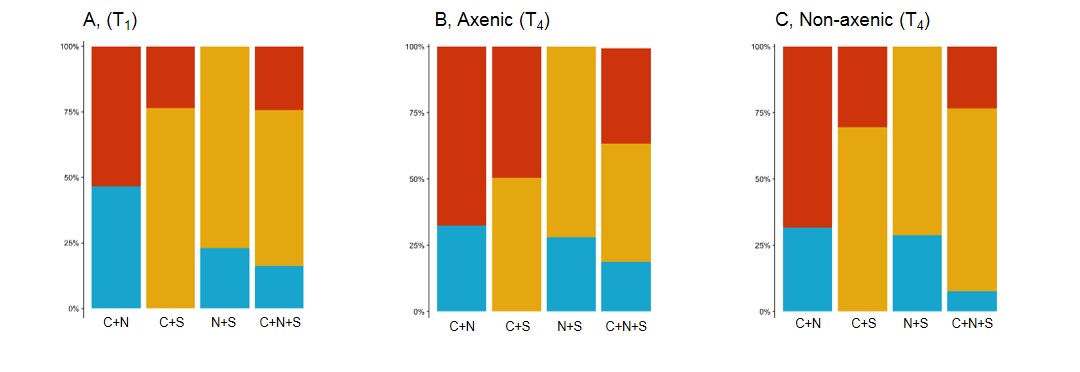

Supplement: FIGURE S3 — The presence of bacteria causes a shift in the relative proportions of diatoms. The relative proportion of C. closterium (red), S. robusta (yellow), and N. phyllepta (blue) grown in pairs or altogether expressed as the number of cells per species to the total number of cells in the well (Left = T1, Center = T4 in axenic conditions, and Right = T4 in non-axenic conditions). Briefly, in axenic conditions an increase in the proportion of C. closterium is observed when grown in the presence of S. robusta, while this observation was not observed in the presence of bacteria. Instead, S. robusta had an increased overall proportion in comparison to C. closterium. [file Image_3.jpg]

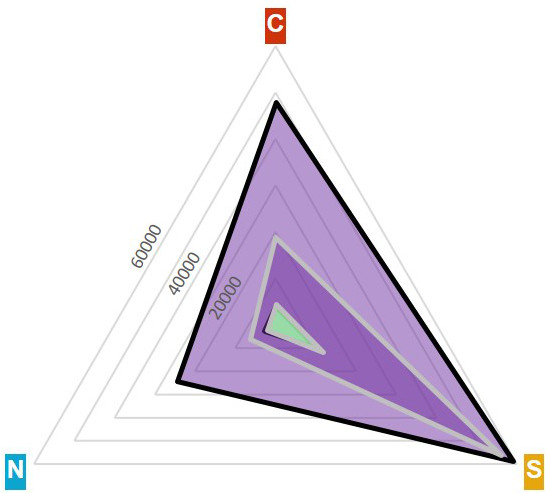

Supplement: FIGURE S4 — The change in the total biovolume (μmł) for each of the three diatom species [C. closterium (red), N. phyllepta (blue), and S. robusta (yellow)] 1 day after inoculation (green area) and 4 days after inoculation (purple area). The gray line indicates the biovolumes under non-axenic conditions while the black line specifies those under axenic conditions. [file Image_4.JPEG]

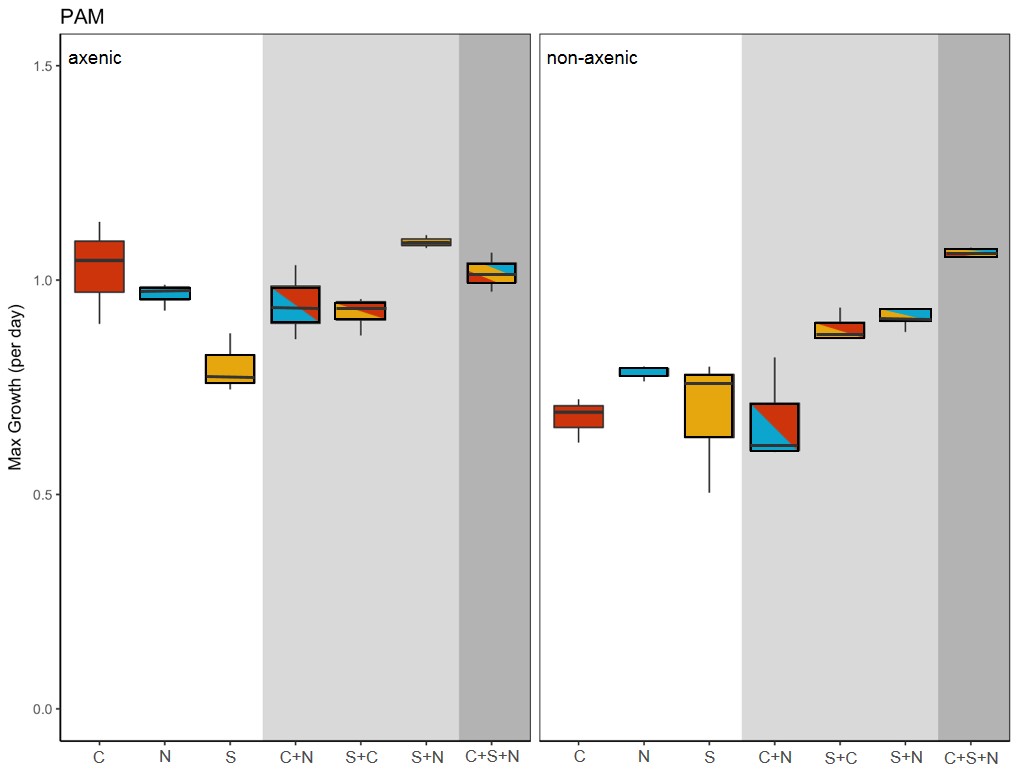

Supplement: FIGURE S5 — PAM measurements depicting the total fluorescence as a proxy for the total maximum growth rates for different combinations of C. closterium ([C], red), N. phyllepta ([N], blue), and S. robusta ([S], yellow) in the presence (non-axenic) or absence of bacteria (axenic). Combinations of multiple diatoms species together have the appropriate colors combined. The white, gray and dark gray background, respectively show diatoms grown in monocultures, diatom pairs or the three diatom-species combined. [file Image_5.JPEG]

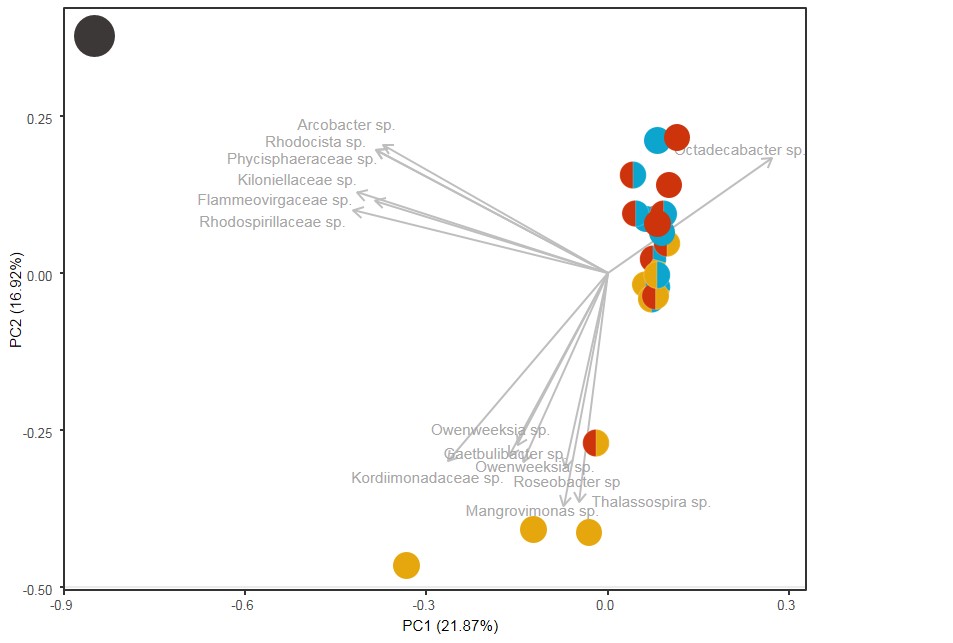

Supplement: FIGURE S6 — PCA on the relative abundance of the bacterial community from the original inoculum (black circle) and when grown in the presence of different combinations of C. closterium (red), N. phyllepta (blue), and S. robusta (yellow). Samples with more diatoms present have circles with the appropriate colors combined. Gray arrows indicate the vectors of the bacterial OTUs with a cumulative loading larger than 30% on the first two axes. The bacterial genus or family is indicated depending on the closest hit. The proportion of the variance explained by each axis is indicated next to that axis. [file Image_6.JPEG]

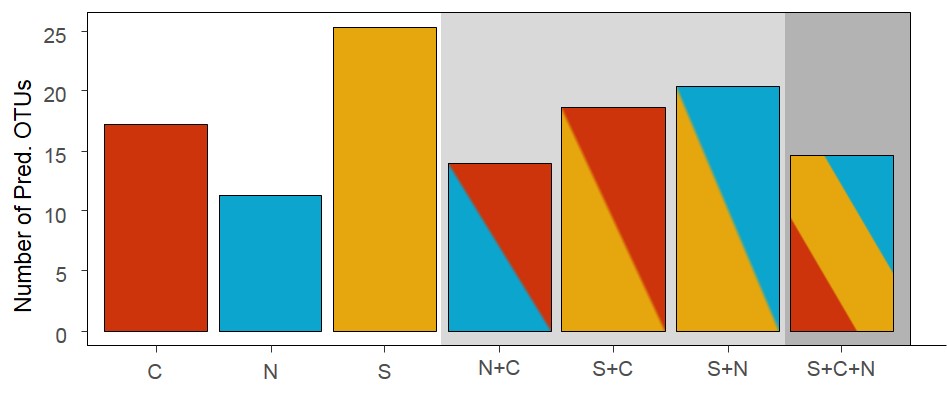

Supplement: FIGURE S7 — Diversity levels of bacteria in the presence of different diatom combinations of C. closterium (C, red), N. phyllepta (N, blue), and S. robusta (S, yellow). Bacterial community diversity was calculated as the average number of predicted OTUs after rarefication of the samples to 781 reads. [file Image_7.JPEG]
